# Academic performance and mental health among Chinese middle and high school students after the lifting of COVID-19 restrictions

**DOI:** 10.3389/fpsyt.2023.1248541

**Published:** 2023-08-14

**Authors:** Hong-Jun Song, Yun-Fei Mu, Cong Wang, Jia Cai, Zhong-Yue Deng, Ai-Ping Deng, Xue-Hua Huang, Xian-Dong Meng, Lan Zhang, Yi Huang, Wei Zhang, Wen-Wu Shen, Jin Chen, Bo Liu, Ru Gao, Jun-Shu Zhao, Mao-Sheng Ran

**Affiliations:** ^1^Mental Health Center, West China Hospital, Sichuan University, Chengdu, Sichuan, China; ^2^West China School of Nursing, Sichuan University, Chengdu, Sichuan, China; ^3^Psychiatric Laboratory, West China Hospital, Sichuan University, Chengdu, Sichuan, China; ^4^West China Hospital, Sichuan University, Chengdu, Sichuan, China; ^5^Department of Clinical Epidemiology and Evidence-Based Medicine, West China Hospital, Sichuan University, Chengdu, Sichuan, China; ^6^Jingzhou Mental Health Center, Jingzhou, Hubei, China; ^7^Wenjiang People’s Hospital, Chengdu, Sichuan, China; ^8^Ya’an Fourth People’s Hospital, Ya’an, Sichuan, China

**Keywords:** academic performance, mental health, students, COVID-19, China

## Abstract

**Background:**

Although the COVID-19 pandemic has greatly changed the way students studied, it is still unknown about the impact of the COVID-19 pandemic on students’ academic performance and mental health.

**Objective:**

To explore the academic performance and mental health status of middle and high school students after the lifting of COVID-19 restrictions in China.

**Methods:**

An online survey was conducted in Sichuan province, China from Dec 14, 2022 to Feb 28, 2023. All participants were students in middle and high schools, recruited via their teachers. The general information, COVID-19-related information, and academic performance were collected. The Patient Health Questionnaire-9 (PHQ-9), Generalized Anxiety Disorder-7 (GAD-7), and Internet Addiction Test (IAT) were used to assess the mental health problems.

**Results:**

Of 60,268 participants, 36,247 (60.2%) middle and high school students reported that their studies were affected by the COVID-19 pandemic, and 24,864 (41.2%) reported that their academic performance had worsened. The prevalence of depression and anxiety symptoms was 38.4 and 32.7%, respectively. There was a significant association between academic performance change and mental health problems. The logistic regression analysis showed that improved academic performance was a protective factor for depression, and declined academic performance was a risk factor for depression and anxiety. Being COVID-19 infected, family members being infected, with quarantine experience, and with COVID-19-related stigma were risk factors for depression and anxiety.

**Conclusion:**

Academic studies and mental health status of middle and high school students in Sichuan, China have been negatively impacted by the COVID-19 pandemic, even after the lifting of COVID-19 restrictions. Students’ academic performance, academic concerns, and mental health status should be considered for educational policymakers and institutions to improve students’ academic studies and mental well-being.

## 1. Introduction

The COVID-19 pandemic had not only posed a great threat to human life and health, but also had an impact on almost all social fields, including students’ studies. In order to stop the spread of the virus, the Chinese government implemented many policies, including business cessation and school closures. To ensure not to interrupt students’ studies, online learning was adopted by educational institutions. Students had to change their way to study from traditional face-to-face learning at school to online learning at home. Previous studies indicated that the shift in learning style during the COVID-19 pandemic had a significant impact on students’ academic performance (1–3) and mental health status (4–7). However, it is still unclear how would academic performance changes affect mental health among Chinese students.

Evidence showed that most of previous studies on academic performance focused on students in colleges or universities, especially medical and nursing students (8–11). However, few studies have been conducted on academic performance in middle and high school students. The relationship between students’ academic performance and mental health should be explored further. China began to lift the COVID-19 restrictions on December 7, 2022. Then, in China, students, including those at middle and high schools, gradually returned back to normal campus life at schools. However, it is still unknown about the students’ academic performance and mental health status, especially after the lifting of COVID-19 restrictions in China.

This study aimed to explore the changes of academic performance and the association between academic performance and mental health among middle and high school students during and after the COVID-19 pandemic in Sichuan Province, China. We hypothesized that academic performance of students was impacted by the COVID-19 pandemic and students’ academic performance was significantly associated with their mental health problems.

## 2. Methods

### 2.1. Study design and participants

From December 14, 2022 to February 28, 2023, the cross-sectional mental health survey was conducted among middle and high school students in Sichuan Province, China. The online self-report questionnaires were sent firstly to school principals or teachers, then these principals and teachers sent the questionnaires directly to their students at schools. Adolescent students voluntarily participated in the online survey via platform of Wenjuanxing. Informed consent was obtained before participants began the survey. This study was approved by the Biomedical Research Ethics Committee of West China Hospital, Sichuan University (NO: 2022–1790).

### 2.2. Measurements

General information: The information included personal information (e.g., gender, age, grade, and ethnic minority, etc.) and family background (e.g., Household register, parents’ marital status, parents’ educational background, monthly family income, family economic level, one-child status, and parenting style, etc.).

COVID-19 related information: This part included whether individuals or family members were infected with COVID-19, quarantine experience, the levels of psychological stress in different stages, the impact of COVID-19 pandemic on daily life routine, and whether their study returned to normal status.

COVID-19 Stigma scale, with good reliability and validity (12), consists of 11 items including “I concern that people infected with novel coronavirus will cause harm to others,” “I will try to distance myself from those infected with novel coronavirus,” etc. Each item is scored from 1–4 (strongly disagree ~ strongly agree) with a total score of 11–44. The cut-off point of 2.5 was used for each item in this study.

Academic performance related information: The impact of COVID-19 on academic performance was assessed by the question “Has your study been affected during COVID-19 pandemic (e.g., worsened, no change, and improved).” This section also included concerns about the impact of the COVID-19 pandemic on their studies, and whether their studies returned to normal status after the lifting of COVID-19 restrictions. The information of online learning was also collected.

The Internet Addiction Test (IAT), including 20 items, was used to assess whether participants were addicted to the internet and its extent (13). The total score ranged from 20 to 100. The cut-off point of 40 was used for internet addiction in this study. IAT is a commonly used test for internet addiction. IAT has good reliability and validity among Chinese populations and young people (14).

The Generalized Anxiety Scale (GAD-7), a commonly used scale, was used to evaluate students’ anxiety symptoms in the last 2 weeks (15). The scale consists of 7 items with a total score ranging from 0 to 21. The cut-off point of 5 was used for mild anxiety. GAD-7 has good reliability and validity among Chinese populations and young people (16–18).^.^

The Patient Health Questionnaire (PHQ-9), a commonly used questionnaire, was used to measure depression symptoms in the last 2 weeks (19). The scale consists of 9 items with a total score range of 0–27. The cut-off point of 5 was used for mild depression. PHQ-9 has good reliability and validity among Chinese populations and young people (20, 21).

### 2.3. Statistical analysis

Participants who reported academic performance being affected by the COVID-19 pandemic and making progress were included in Group 1. Those who reported academic performance being same as before were included in Group 2. Those who reported academic performance being affected by the COVID-19 pandemic and being declined were included in Group 3. The SPSS 25.0 was applied to analyze the data. Descriptive statistics were used to analyze general information. Correlation analysis was used to explore the variables associated with academic performance. ANOVA and post-hoc test were used to analyze differences in mental health problems among the three groups. The logistic regression was used to explore both risk and protective factors for depression and anxiety. Factors that showed a significant level in univariate analysis were entered into the logistic regression analysis. As this study mainly focused on the relationship between risk factors and mental health problems rather than predicting individual mental health problems, therefore, the good fitness of the logistic regression model (e.g., R-squared and Hosmer-Lemeshow test) was not of significant concern. Statistical significance was taken as *p* < 0.05.

## 3. Results

### 3.1. General information

Table 1 shows the general information of the participants. A total of 60,268 participants voluntarily participated in this study and finished the questionnaires from middle and high schools in Sichuan, China. In total, all these middle and high school students aged from 11 to 19 (15.009 ± 1.813) years were included in the study. Among them, 27,459 (45.6%) participants were male, 53,459 (88.7%) were Han Chinese, 49,819 (82.7%) were resident in rural areas and 15,016 (24.9%) were from single-child family. There were 36,111 (59.9%) students from senior high schools, and 12,422 (20.6%) students were facing graduation. The proportion of students infected with COVID-19 was 41.6%.

**Table 1:** Demographic characteristics and academic performance among participants.

	Total *N* (%)	Group 1 *N* (%)	Group 2 *N* (%)	Group 3 *N* (%)	*X* ^2^	*p*
Total: *N* (%)		11,383 (18.9)	24,021 (39.9)	24,864 (41.2)		
Gender						*p* < 0.001
Male	27,459 (45.6)	5,912 (51.9)	10,718 (44.6)	10,829 (43.6)	235.590	
Ethnicity					88.122	*p* < 0.001
Han	53,459 (88.7)	10,171 (89.4)	21,588 (89.9)	21,700 (87.3)		
Other ethnic groups	6,809 (11.3)	1,212 (10.6)	2,433 (10.1)	3,164 (12.7)		
Age (year)					202.161	*p* < 0.001
≤13	14,642 (24.3)	2,898 (25.5)	6,373 (26.5)	5,371 (21.6)		
14–17	41,610 (69.0)	7,729 (67.9)	16,249 (67.6)	17,632 (70.9)		
≥18	4,016 (6.7)	756 (6.6)	1,399 (5.8)	1861 (7.5)		
Household registration					85.838	*p* < 0.001
Rural	49,819 (82.7)	9,744 (85.6)	19,739 (82.2)	20,336 (81.8)		
Urban	10,449 (17.3)	1,639 (14.4)	4,282 (17.8)	4,528 (18.2)		
Grade					352.561	*p* < 0.001
Junior high school	24,157 (40.1)	4,775 (41.9)	10,323 (43.0)	9,059 (36.4)		
Senior high school	36,111 (59.9)	6,608(58.1)	13,698 (57.0)	15,805 (63.6)		
Graduating class	12,422 (20.6)	2,393 (21.0)	4,774 (19.9)	5,255 (21.4)	13.318	*p* = 0.001
Family economic level					563.690	*p* < 0.001
> average	1,362 (2.3)	395 (3.5)	501 (2.1)	466 (1.9)		
Average	41,785 (69.3)	8,209 (72.1)	17,442 (72.6)	16,134 (64.9)		
< average	17,121 (28.4)	2,779 (24.4)	6,078 (25.3)	8,264 (33.2)		
Single-child	15,016 (24.9)	2,922 (25.7)	6,056 (25.2)	6,038 (24.3)	9.884	*p* = 0.007
Parent divorced	11,699 (19.4)	2,120 (18.7)	4,625 (19.3)	4,954 (19.9)	8.886	*p* = 0.012
Parenting style					806.867	*p* < 0.001
Authoritative	34,207 (56.8)	7,532 (66.2)	13,894 (57.8)	12,781 (51.4)		
Authoritarian	13,672 (22.7)	2,184 (19.2)	5,396 (22.5)	6,092 (24.5)		
Neglectful	3,844 (6.4)	425 (3.7)	1,395 (5.8)	2024 (8.1)		
Permissive	8,545 (14.2)	1,242 (10.9)	3,336 (13.9)	3,967 (16.0)		
Infected with COVID-19 (Confirmed / suspected)					915.842	*p* < 0.001
Yes	25,073 (41.6)	3,673 (32.3)	9,409 (39.2)	11,991 (48.2)		
Family members infected with COVID-19 (Confirmed / suspected)					998.628	*p* < 0.001
Yes	27,493 (45.6)	3,986 (35.0)	10,502 (43.7)	13,005 (52.3)		
Quarantine experience	15,788 (26.2)	3,117 (27.4)	5,948 (24.8)	6,723 (27.0)	42.997	*p* < 0.001
COVID-19 related stigma	15,223 (25.3)	3,987 (35.0)	5,671 (23.6)	5,565 (22.4)	718.865	*p* < 0.001
Worrying about academic performance due to COVID-19					1424.883	*p* < 0.001
Yes	32,561 (54.0)	5,503 (48.3)	11,354 (47.3)	15,704 (63.2)		
Daily life routine					7094.238	*p* < 0.001
No change	27,484 (45.6)	7,140 (62.7)	13,834 (57.6)	6,510 (26.2)		
Partly impacted	28,465 (47.2)	3,887 (34.1)	9,365 (39.0)	15,213 (61.2)		
Seriously impacted	4,319 (7.2)	356 (3.1)	822 (3.4)	3,141 (12.6)		
Regular exercise	34,119 (56.6)	8,894 (78.1)	13,677 (56.9)	11,548 (46.4)	3194.039	*p* < 0.001

Group 1, participants with academic performance progress; Group 2, participants with academic performance unaffected; Group 3, participants with academic performance declined.

### 3.2. Academic performance

For the academic performance, 11,383 (18.9%) participants reported that they had achieved academic progress, 24,021 (39.9%) had the same as before, and 24,864 (41.2%) were affected negatively by the COVID-19 pandemic and their academic performance declined. Participants in academic performance progress group (G1) had significantly higher rates of being male (51.9%), household registered in rural area (85.6%), with higher monthly family income (3.5%), authoritative parenting style (66.2%), and with regular exercise (78.1%) than those in G2 (44.6, 82.2, 2.1, 57.8, 56.9%) and G3 (43.6, 81.1, 1.9, 51.4, 46.4%) (*p* < 0.001). The rates of students with COVID-19-related stigma in participants with academic performance progress (G1) (35.0%) was significantly higher than that in G2 and G3 (23.6, 22.4%) (*p* < 0.001).

Compared with Group 1 and Group 3, participants in academic performance unaffected group (Group 2) had the lowest rates of being in graduating class (19.9% vs. 21.0, 21.4%), with quarantine experience (24.8% vs. 27.4, 27.0%) and worrying about the impact of COVID-19 pandemic on their studies (47.3% vs. 48.3, 63.2%) (*p* ≤ 0.001), and the highest rates of being Han Chinese (89.9% vs. 89.4, 87.3%), being in junior middle school (43.0% vs. 41.9, 36.4%) (*p* < 0.001). Participants’ worsen academic performance was significantly associated with household registered in urban area (18.2% vs. 14.4, 17.8%), in minority ethnic group (12.7% vs. 10.6, 10.1%), being senior high school students (63.6% vs. 58.1, 57.0%), lower family income (33.2% vs. 24.4, 25.3%), infected with COVID-19 (48.2% vs. 32.3, 39.2%), and family members infected (52.3% vs. 35.0, 43.7%) (*p* ≤ 0.001).

### 3.3. Academic performance and mental health

Table 2 shows the differences of mental health problems among three groups. The prevalence of depression symptoms, anxiety symptoms, and internet addiction among middle and high school students were 38.4, 32.7, and 58.1%, respectively. The rates of depression symptoms and anxiety symptoms were gradually increased from Group 1 to Group 3 (*p* < 0.001). The rates of internet addiction were 48.1, 50.9, and 69.8% in three groups, and were also gradually increased (*p* < 0.001). Participants with declined academic performance had the highest rates of mental health problems among the three groups (*p* < 0.001).

**Table 2:** Differences of mental health problems among participants in three groups.

	Total *N* (%)	Group 1 *N* (%)	Group 2 *N* (%)	Group 3 *N* (%)	F/X^2^	*p*	Post-hoc
Depression (PHQ-9)					1530.205	*p* < 0.001	
≥5	23,142 (38.4)	3,305 (29.0)	8,039 (33.5)	11,798 (47.5)			
Anxiety (GAD-7)					1130.102	*p* < 0.001	
≥5	19,677 (32.7)	2,987 (26.2)	6,674 (27.8)	10,016 (40.3)			
Internet addiction (IAT)					2384.684	*p* < 0.001	
≥40	35,034 (58.1)	5,470 (48.1)	12,214 (50.9)	17,350 (69.8)			
Depression: PHQ-9 mean score	4.76 ± 6.14	3.73 ± 5.98	4.16 ± 5.78	5.82 ± 6.39	637.530	*p* < 0.001	G1 < G2 < G3
Anxiety: GAD-7 mean score	3.52 ± 5.03	2.81 ± 4.83	3.01 ± 4.70	4.33 ± 5.30	544.715	*p* < 0.001	G1 < G2 < G3
Internet addiction: IAT mean score	44.75 ± 17.07	41.48 ± 18.49	42.00 + 15.92	48.90 ± 16.59	1317.055	*p* < 0.001	G1 < G2 < G3

Group 1, participants with academic performance progress; Group 2, participants with academic performance unaffected; Group 3, participants with academic performance declined.

**Figure 1:**
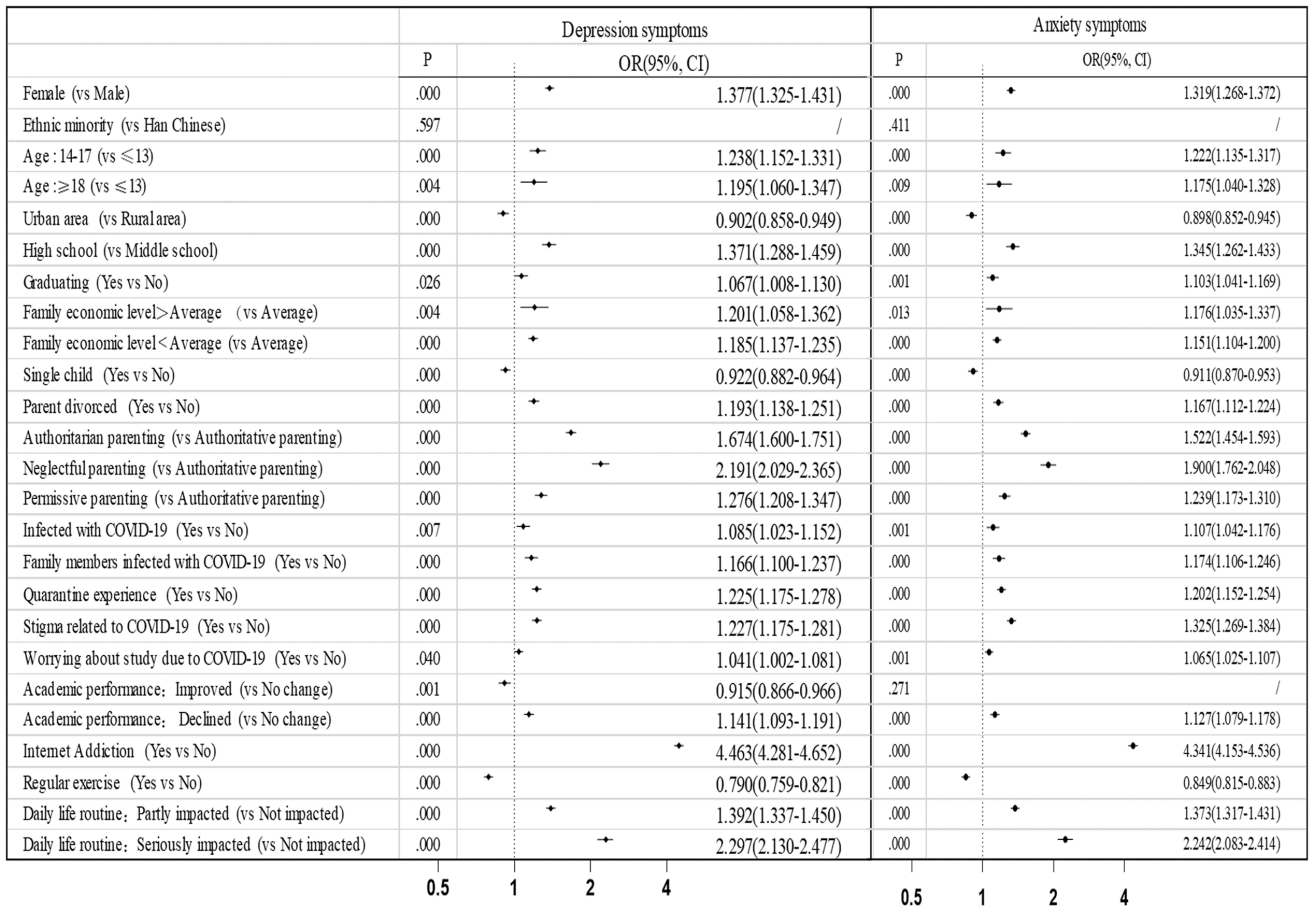
The factors associated with depression and anxiety.

The results of logistic regression showed that being female, aged 14–17 and 18 years above, with parents divorced were risk factors for depression (OR = 1.377, 1.238, 1.195, 1.193) and anxiety (OR = 1.319, 1.222, 1.175, 1.167) (*p* < 0.05) (Figure 1). Participants being only child in one-child family, household registered in urban area, and with regular exercise had lower risk of depression (OR = 0.922, 0.902, 0.790) and anxiety (OR = 0.911, 0.898, 0.849) (*p* < 0.001). Participants in high-income and low-income families had a significantly higher risk of depression (OR = 1.201, 1.185) and anxiety (OR = 1.176, 1.151) than those from middle-income families (*p* < 0.05). Being infected of COVID-19, family members being infected, with quarantine experience, with COVID-19-related stigma were risk factors for depression (OR = 1.085, 1.166, 1.225, 1.227) and anxiety (OR = 1.107, 1.174, 1.202, 1.325) (*p* < 0.05).

In terms of academic-related factors, being high school students, facing graduation, concerns about the impact of COVID-19 on academic performance were risk factors for depression (OR = 1.371, 1.067, 1.041) and anxiety (OR = 1.345, 1.103, 1.065) (*p* < 0.05). Participants with declined academic performance had 1.141 and 1.127 times higher risk of depression and anxiety than those without change of academic performance, and improved academic performance was a protective factor for depression (*p* ≤ 0.001).

## 4. Discussion

This is the first study on the relationships between academic performance and mental health problems among middle and high school students after the lifting the COVID-19 restrictions in China. This study explored the characteristics of students’ learning style and academic performance, and the relationship between academic performance and mental health problems. The results of this study showed that 36,247 (60.2%) middle and high school students’ studies had been affected by the COVID-19 pandemic, and 24,864 (41.2%) students reported a declined academic performance. The rate of declined academic performance in this study is much higher than that in a previous study that 11.6% of high achieving students reported decline in academic performance after the COVID-19 outbreak (3). The shift in learning style (e.g., online learning and hybrid learning) may be one of the reasons (22–24). The results of this study showed that Han Chinese was associated with academic performance unaffected, and minority ethnical group was significantly related to academic performance declined. The possible reasons may be related to different social capital, vulnerability, and learning style (22, 25, 26).

The results of this study identified that the rates of depression and anxiety symptoms of middle and high school students were 38.4 and 32.7%, which is different with a previous study (27). The possible reason may be related to the different time of the investigations. The results of this study also showed that being female, household registered in rural area, studying at high school, and with siblings were risk factors for depression and anxiety symptoms, which is consistent with previous studies (27–32). This study indicates that the declined academic performance is a risk factor and the improved academic performance is a protective factor for depression and anxiety, which has tested the research hypothesis on the association between academic performance and mental health problems. The possible reasons maybe include the followings. First, lower grades and test may be associated with high levels of psychological distress (33), which may lead to mental health problem (34, 35). Second, students with high achievement might be more likely to adjust the environment and coping with negative emotion (36). Further studies should be conducted to explore the relationship betweem academic performance and mental health problems.

The results of this study showed that the prevalence of internet addiction was 58.3% among middle and high school students, which is higher than that in previous studies (37, 38). This study suggests that internet addiction is negatively associated with students’ academic performance, which is consistent with previous studies (39) and has tested the research hypothesis. Additionally, this study identified that students with internet addiction had a significantly higher risk of depression and anxiety symptoms, which is also consistent with previous studies (40). Therefore, it is crucial for education ministry to develop new policies and guidelines to prevent internet addiction for improving mental wellbeing among middle and high school students. Further studies should be conducted in this important area.

The results of this study showed that declined academic performance was significantly associated with being infected with COVID-19, family members infected with COVID-19, worrying about academic performance due to COVID-19, and daily lift routine disturbed by COVID-19. Previous studies showed that students with someone closed being infected of COVID-19 and with psychological distress had higher risks of poor academic performance (41, 42). The results of this study also showed that factors related to COVID-19 pandemic were also positively associated with depression and anxiety symptoms, which is consistent with previous studies (30, 32, 43–45). Moreover, COVID-19-related stigma was risk factors of mental health problems (e.g., depression and anxiety symptoms) (46). Education and health institutions should consider these potential factors for development of educational and health policies and psychosocial interventions for improving mental wellbeing of students.

The results of this study showed regular exercise was associated with academic performance progress, and was protective factor for depression and anxiety. Previous studies have suggested that sufficient physical activities could improve learning efficacy (47), reduce the risk of mental health problems, and improve academic performance (42, 48–50). Education institution should help students to establish regular exercise habits to improve students’ mental health status.

## 5. Strengths and limitations

This study had a few strengths. To our knowledge, this study should be the first study to explore the changes in academic performance and the association between academic performance and mental health problems among middle and high school students after the lifting of COVID-19 restrictions in China. It is possible for this study to explore the impact of home-based online learning on students’ academic performance. This study was conducted via school teachers (a questionnaire was distributed from school teachers to their students directly) in middle and high schools in Sichuan Province, China, and the quality of the results should be high.

This study had a few limitations. First, this study is a cross-sectional design, and no causal associations should be inferred. Further long-term follow-up studies should be conducted in this area to explore the causal relationship. Second, this study was conducted only in Sichuan province, China, and the results may not be generalized to other areas with different situations.

## 6. Conclusion

The objective of this study was to explore the changes of academic performance and associations between academic performance and mental health problems after the lifting of COVID-19 restrictions among middle and high school students in Sichuan, China. This study indicates a severe change of students’ academic performance as the impact of COVID-19 pandemic, and a significant association between students’ academic performance and mental health problems (e.g., symptoms of depression, anxiety, and internet addiction). Improved academic performance was a protective factor for depression, and declined academic performance, concerns about the COVID-19 impact and graduation were risk factors for both depression and anxiety. It is crucial for education and health institutions to consider these potential factors for development of educational and health policies and psychosocial interventions, and improve mental wellbeing of middle and high school students.

## Data availability statement

The original contributions presented in the study are included in the article/supplementary material, further inquiries can be directed to the corresponding author.

## Ethics statement

The studies involving humans were approved by Biomedical Research Ethics Committee of West China Hospital, Sichuan University. The studies were conducted in accordance with the local legislation and institutional requirements. Written informed consent for participation in this study was provided by the participants’ legal guardians/next of kin.

## Author contributions

M-SR designed this study. M-SR, H-JS, Y-FM, JiaC, CW, Z-YD, A-PD, YH, WZ, W-WS, and JinC conducted this study. H-JS, Y-FM, and M-SR conducted data analysis and wrote the first draft of the paper. H-JS, Y-FM, CW, JiaC, Z-YD, A-PD, X-HH, X-DM, LZ, YH, WZ, W-WS, JinC, BL, RG, J-SZ, and M-SR participated in the data collection and made contributions to critical revision of the manuscript. All authors contributed to the article and approved the submitted version.

## Funding

This work was funded by Initial Research Fund, West China Hospital (WCH, No: 136220012, PI: M-SR). The funder had no role in the design and conduct of the study, collection, management, analysis, and interpretation of the data; preparation, review, or approval of the manuscript; and decision to submit the manuscript for publication.

## Conflict of interest

The authors declare that the research was conducted in the absence of any commercial or financial relationships that could be construed as a potential conflict of interest.

## Publisher’s note

All claims expressed in this article are solely those of the authors and do not necessarily represent those of their affiliated organizations, or those of the publisher, the editors and the reviewers. Any product that may be evaluated in this article, or claim that may be made by its manufacturer, is not guaranteed or endorsed by the publisher.
